# Genome Sequences of Potential Probiotic *Lactobacillus rhamnosus* Isolates from Human Infants

**DOI:** 10.1128/genomeA.00107-17

**Published:** 2017-04-06

**Authors:** Jason W. Arnold, Andrea Monteagudo-Mera, Eric Altermann, Maria Belen Cadenas, Amanda L. Thompson, M. Andrea Azcarate-Peril

**Affiliations:** a Division of Gastroenterology and Hepatology and Microbiome Core Facility, Department of Medicine, Center for Gastrointestinal Biology and Disease, University of North Carolina, Chapel Hill, North Carolina, USA; b AgResearch Ltd., Palmerston North, New Zealand; c Departments of Anthropology and Nutrition, University of North Carolina, Chapel Hill, North Carolina, USA; d Riddet Institute, Massey University, Palmerston North, New Zealand

## Abstract

Probiotics provide health benefits to their hosts, including modulation of host immune response, inhibition of colonization by pathogens, modulation of the gut microbiota, and epithelial barrier enhancement. Here, we present the draft genome sequences of two newly isolated *Lactobacillus rhamnosus* strains of probiotic potential from healthy human infants.

## GENOME ANNOUNCEMENT

Probiotics are live microbes that confer a benefit to their hosts when administered in adequate amounts (1). Support for the use of probiotics as interventions for gastrointestinal diseases is increasing (2–4). Most of the diversity of the gut microbiome exists at the strain level (5), calling for more in-depth studies of individual bacterial strains to assess phenotypes that could have a beneficial impact. *Lactobacillus rhamnosus* is a lactic acid bacterium with probiotic properties when associated with the host gut (6) or skin (7). Here, we report the genome sequences of two novel strains of *L. rhamnosus* isolated from stool samples from healthy human infants (8).

We isolated 175 bacterial strains from stool samples from healthy infants (0 to 2 years old) (8). Each strain was initially isolated by plating stool samples from healthy human infants on de Man, Rogosa, and Sharpe (MRS) agar plates (Hardy Diagnostics, Santa Maria, CA) under anaerobic conditions, separating individual colonies. Subsequently, single colonies were selected and grown in MRS broth. Phylogenetic identification of isolates was performed by sequencing of the 16S rRNA gene, and selected isolates were further characterized by random amplification of polymorphic DNA (RAPD) (9, 10) to identify unique strains. Two novel *L. rhamnosus* isolates (AMC010 and AMC143) were identified in this study.

Genomic DNA from AMC010 and AMC143 was isolated using UltraClean microbial DNA isolation kit (Mo Bio). AMC010 was subjected to sequencing on the Ion Torrent PGM platform (Life Technologies, Inc.), generating sequence data of 485.3 Mb over 3,022,036 reads, with an average read length of 160 bp, providing 43× coverage of the 3.14-Mb genome. AMC143 genomic DNA (gDNA) was sequenced on the Roche FLX Titanium platform, generating 69.4 Mb of sequence over 318,612 reads, with an average read length of 210 bp, providing 23× coverage of the 2.87-Mb genome. Raw reads were quality filtered and assembled using Newbler (Roche) (11). Assembled contigs were annotated using an updated version of the GAMOLA annotation pipeline (12).

AMC010 has a G+C content of 46.6%, 53 annotated tRNA genes, and 3,171 annotated coding sequences (CDSs), with 162 unique genes compared to other sequenced and annotated *L. rhamnosus* genomes using the EDGAR genome analysis software (13). In addition to uncharacterized hypothetical proteins, AMC010 contains a unique toxin-antitoxin pair and unique bacteriophage components. AMC143 has a G+C content of 46.6%, 47 annotated tRNA genes, and 2,835 annotated CDSs, with 73 unique genes compared to other sequenced and annotated *L. rhamnosus* genomes. Included among these unique genes are bacteriophage components, a phosphotransferase (PTS) fructose transporter system, PTS lactose transporter system, and a large number of previously uncharacterized hypothetical proteins.

### Accession number(s).

This whole-genome shotgun project has been deposited at DDBJ/ENA/GenBank under accession numbers MSTB00000000 (AMC143) and MSTC00000000 (AMC010).
